# 11β-Hydroxysteroid Dehydrogenase Type 1 as a Potential Treatment Target in Cardiovascular Diseases

**DOI:** 10.3390/jcm11206190

**Published:** 2022-10-20

**Authors:** Daria Kupczyk, Renata Studzińska, Renata Kołodziejska, Szymon Baumgart, Martyna Modrzejewska, Alina Woźniak

**Affiliations:** 1 Department of Medical Biology and Biochemistry, Faculty of Medicine, Collegium Medicum in Bydgoszcz, Nicolaus Copernicus University in Toruń, Karłowicza 24, 85-092 Bydgoszcz, Poland; 2 Department of Organic Chemistry, Faculty of Pharmacy, Collegium Medicum in Bydgoszcz, Nicolaus Copernicus University in Toruń, Jurasza 2, 85-089 Bydgoszcz, Poland

**Keywords:** 11β-hydroxysteroid dehydrogenase, glucocorticoids, metabolic syndrome, cardiovascular diseases, 11β-HSD1 selective inhibitors

## Abstract

Glucocorticoids (GCs) belong to the group of steroid hormones. Their representative in humans is cortisol. GCs are involved in most physiological processes of the body and play a significant role in important biological processes, including reproduction, growth, immune responses, metabolism, maintenance of water and electrolyte balance, functioning of the central nervous system and the cardiovascular system. The availability of cortisol to the glucocorticoid receptor is locally controlled by the enzyme 11β-hydroxysteroid dehydrogenase type 1 (11β-HSD1). Evidence of changes in intracellular GC metabolism in the pathogenesis of obesity, metabolic syndrome (MetS) and cardiovascular complications highlights the role of selective 11β-HSD1 inhibition in the pharmacotherapy of these diseases. This paper discusses the role of 11β-HSD1 in MetS and its cardiovascular complications and the importance of selective inhibition of 11β-HSD1.

## 1. Introduction

The basic carbohydrate used in human metabolism is glucose, which is a universal energy material for all cells. There are a number of factors that influence the homeostasis of this carbohydrate metabolism. One of them is the effect of insulin-antagonistic hormones. Glucocorticoid (GC) hormones have such an effect, which is why they have a significant impact on glucose metabolism [1]. In physiological amounts, they are necessary for the regulation of a number of vital physiological functions of the body, such as reproduction, growth, metabolism, fat distribution, immune and inflammatory reactions, the functioning of the cardiovascular system, the central nervous system, and the maintenance of water and electrolyte balance. However, their excess induces pathological processes, such as intensification of diabetogenic effects or deterioration of metabolic control in people with previously diagnosed diabetes [2]. The excess of GCs results, among others, in development of arterial hypertension, which is one of the most common diseases of the cardiovascular system. Excessive cortisol production occurring, among others, in Cushing’s syndrome or metabolic syndrome (MetS), leads to a number of systemic changes resulting in the redistribution of adipose tissue, arterial hypertension, carbohydrate disorders, dyslipidemia and coagulopathies [3]. All of these pathologies increase the risk of cardiovascular episodes up to four times. Untreated Cushing’s disease significantly increases the cardiovascular risk and limits the treatment options for secondary organ complications. Mortality from cardiovascular disease (heart attack, heart failure, stroke) in people with Cushing’s disease is five times higher than in the general population [4]. GCs act through the intracellular glucocorticoid receptor (GR), and the enzyme that locally controls the availability of their active form to this receptor is 11β-hydroxysteroid dehydrogenase (11β-HSD1) isoform 1. Thus, 11β-HSD1 may be a potential biomarker in cardiovascular diseases because the increase in its activity translates into an increase in the level of GCs in the body, whereas selective inhibition of this enzyme may prove to be a new target in drug research. Focusing research on the search for 11β-HSD1 inhibitors could provide valuable information in the search for a drug that could be used in the treatment of type 2 diabetes and its cardiovascular complications. In the event of obtaining positive results, it would mean the possibility of using rational and effective therapy [5].

## 2. Glucocorticoids and Their Role in the Body

**Figure 1:**
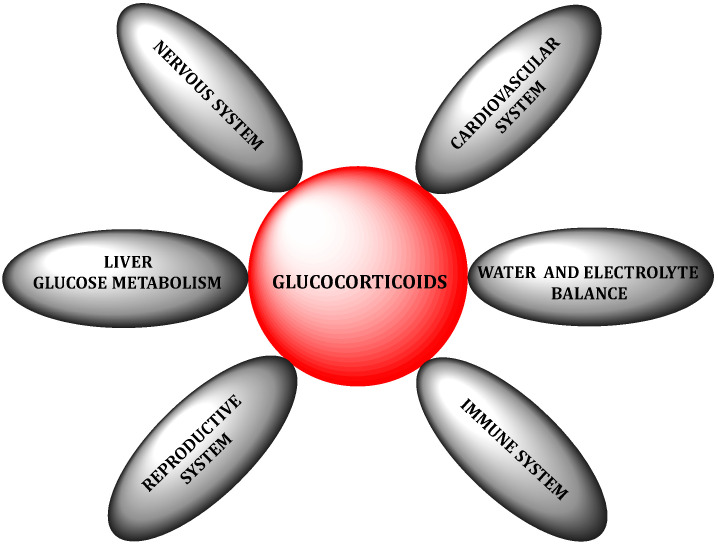
Role of glucocorticoids in physiological processes.

**Figure 2:**
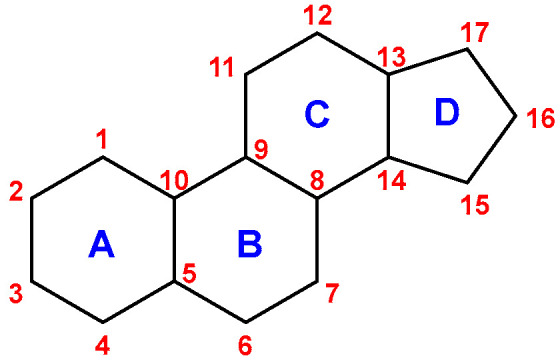
The structure of steroid nucleus.

Glucocorticoids, the main representative of which in humans is cortisol (95% of glucocorticoid activity), play a significant role in the regulation of many physiological processes and ensure the maintenance of homeostasis in the body under stress (Figure 1) [6]. They play an important role in the metabolism of carbohydrates, fats and proteins. They take part in the regulation of water and electrolyte balance. They are responsible for the distribution of adipose tissue. They also affect the functioning of the central nervous system and the cardiovascular system. They take part in the regulation of immunological and inflammatory processes [7,8,9,10]. The structure of GCs is based on the hexadecahydro-*1H*-cyclopenta[a]phenanthrene ring, also known as the “steroid nucleus”, which is the basic structure of androgens, estrogens and progestins [11] (Figure 2).

The precursor to the synthesis of GCs is cholesterol, which mainly comes from circulating low-density lipoproteins (LDL) in the plasma [12]. GCs are secreted by the cortex layer of the adrenal glands. The regulation of endogenous GC secretion takes place under the control of the hypothalamic–pituitary–adrenal axis (HPA) on the basis of negative feedback, and the main factor controlling cortisol secretion is adrenocorticotropic hormone (ACTH) [13]. By responding to a number of stimuli (physical, emotional and circadian), the hypothalamus activates the pathway that leads to the synthesis of GCs. 

Then, there is the secretion of hypothalamic neurohormones, which stimulate the production of the proopiomelanocortin peptide, from which the ACTH entering the blood is proteolytically derived from proopiomelanocortin peptide. This hormone, acting on the adrenal cortex, initiates the synthesis of cortisol. Thus, changes in plasma cortisol are closely related to changes in ACTH levels [14,15]. There is a pulsatile release of ACTH and cortisol. The release of cortisol occurs in a circadian rhythm, i.e., increases at night between 3–5 h of sleep with the peak of secretion in the first hour after awakening, and then decreases. During the day, there are a few more secretory impulses that are associated with physical effort or food intake. The lowest cortisol levels are observed in the late evening hours and in the first hours of sleep [16,17,18]. Of course, the circadian rhythm of cortisol secretion is subject to individual variability, e.g., due to changes in sleep hours, meals or stress [19]. Approximately 70–90% of GCs are transported in plasma bound to proteins by corticosteroid-binding globulin (CBG), called transcortin [20]. GCs affect the cells of the human body through specific intracellular GR, located in the cytoplasm, cell nucleus and the cell membrane [21]. The human GC receptor is a protein composed of three domains and belongs to the nuclear hormone receptor superfamily, which includes, among others, receptors for vitamin D or thyroid hormones and receptors for other steroids (androgens, estrogens, progestins, and mineralocorticoids) [22]. In turn, in the non-genomic mechanism of GC action, secondary messengers (calcium ions, diacylglycerol, cGMP, cAMP, and inositol triphosphate) are activated, activating signaling pathways dependent on them [23]. GCs increase blood glucose levels by inhibiting peripheral glucose uptake by muscles and adipose tissue. They intensify the process of gluconeogenesis in the liver by stimulating the activity of glucose-6-phosphatase and phosphoenolpyruvate carboxykinase. They indirectly intensify the process of glycogenolysis. By decreasing peripheral glucose uptake, they increase insulin secretion, which causes the development of insulin resistance. Their chronic excess may lead to the development of pre-diabetes or diabetes [24,25]. In adipose tissue, GCs show a lipolytic effect, through the release of free fatty acids, their burning in the liver and the production of ketone bodies; they also stimulate the accumulation of visceral fat [26]. It is cortisol that affects the preadipocytes, as it accelerates their differentiation into adipocytes [27]. It also influences the expression of genes of mature fat cells, causing their hypertrophy and leading to the accumulation of lipids. Excess body fat and its uneven deposition around the face, torso, neck and abdomen are symptoms of excess GCs [28]. This leads to an increase in triglycerides stored in the liver. 

Moreover, as a result of excessive stimulation of the GC receptor, excess GCs may lead to the development of dyslipidemia due to the inhibition of lipoprotein lipase activity in adipose tissue [29]. 

In type 2 diabetes, associated with obesity and other components of MetS, a condition known as functional hypercortisolemia develops due to overstimulation of corticotropin-secreting cells in the hypothalamus [30,31]. In addition, the major metabolic effects of GCs may be related to the effects of cortisol on AMP-activated protein kinase (AMPK) activity. This enzyme plays an important role in glucose and lipid homeostasis. Thus, changes in its activity under the influence of GCs could explain, i.a., lipid deposition in visceral fat and changes in the heart [32]. The metabolism of steroids and conjugation with the rest of the glucuronic and sulfuric acids lead to inactivation of GCs and increase their solubility in water, thus facilitating excretion in the urine. The liver is the primary site for conversion of GCs to inactive metabolites and conjugation. Approximately 90% of the resulting metabolites are excreted by the kidneys [33]. The enzyme responsible for the mutual conversion of biologically active cortisol to inactive cortisone is 11β-hydroxysteroid dehydrogenase [34].

## 3. 11β-HSD-Isoforms and Their Role in the Proper Functioning of the Organism

**Figure 3:**
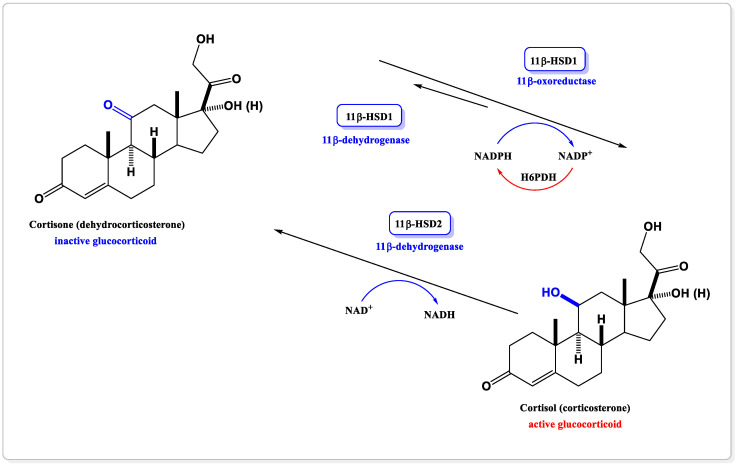
The physiological role of the two isoforms of 11β-HSD.

11β-hydroxysteroid dehydrogenase belongs to the short-chain family of NAD- or NADP-dependent dehydrogenases/reductases (SDRs) [34,35]. At the end of the 1990s, two isoforms of this enzyme, 11β-HSD1 and 11β-HSD2, were described [36]. Two isozymes of 11β-hydroxysteroid dehydrogenase catalyze the interconversion between inactive glucocorticoids (human cortisone and rodent 11-dehydrocorticosterone) and hormonally active glucocorticoids (human cortisol and rodent corticosterone) (Figure 3). The biological activity of the glucocorticoid molecule is regulated by enzymatic modification of the substituent at the C11 position. These transformations include oxidation and reduction reactions within the cortisol/corticosterone hydroxyl group and the cortisone/dehydrocorticosterone carbonyl group, respectively [34,37].

Both isoforms are microsomal enzymes localized to the endoplasmic reticulum cell membrane, but they are encoded by separate genes and differ in function, distribution in the body, and cofactor and substrate affinity—Table 1. 11β-HSD1 is a product of the HSD11B1 gene on chromosome 1 both in humans and in rodents, while 11β-HSD2 is encoded by a gene located on chromosome 16 in human and 8 in rodents [38,39]. Isoform 1, also called hepatic, occurs in tissues rich in the glucocorticoid receptor, mainly in the liver, brain, pancreatic islets, and adipose tissue, as well as lungs, gonads and bones [40,41,42,43]. In vitro, 11β-HSD1 is the enzyme that has a bidirectional activity, shows the activity of NADP^+^-dependent 11β-dehydrogenase in the oxidation reaction and NADPH-dependent 11-oxoreductase in the reduction reaction [44]. 11β-HSD1 acts in vivo mainly as a reductase, thereby activating GCs from circulating 11-oxo precursors to the respective 11βOH receptor ligands [34,42]. In most tissues, it is co-expressed in the endoplasmic reticulum with hexose-6-phosphate dehydrogenase (H6PDH), which generates NADPH requisite for reductase activity [34,37]. Isoform 2, called kidney, acts only unidirectionally, as NAD^+^-dependent dehydrogenase. Compared to 11β-HSD1, it is expressed in tissues rich in the mineralocorticoid receptor (MR), mainly in the kidneys, large intestine, salivary glands, and placenta, and is characterized by a much higher affinity for cortisol (100 times greater than 11β-HSD1) [44,45].

11β-HSD enzymes are classically regarded as regulators of GC function at the cell/tissue level, so they are essential in maintaining proper mineral and glucocorticoid metabolism [34,46,47]. By modulating the levels of GCs in various tissues, these enzymes play an important role in many physiological processes, including carbohydrate metabolism, memory processes, obesity, blood vessel reactivity, maintenance of normal blood pressure and osteoporosis [48]. The basic role of 11β-HSD1 is to increase the concentration of the active form of GCs in the tissue and, consequently, to activate the glucocorticoid receptor. Thus, it regulates the physiological functions of GCs, e.g., participation in hepatic gluconeogenesis, as well as the processes taking place in adipose tissue [34,45,48,49]. The role of 11β-HSD1 includes both “endocrine” regulation of circulating corticosteroid availability and systemic GC exposure, and fine-tuning local tissue and cell specific exposure through “intracrine” activation of cortisol independent of circulating cortisol [50]. The regulation of systemic endocrine cortisol activation is mainly determined by hepatic 11β-HSD1, which is constitutively and extensively expressed in the liver [50,51,52]. In contrast, regulation of 11β-HSD1 in tissues such as adipose, muscle, bone and sites of inflammation is dynamically regulated in a highly cellular and context-specific manner [50,53,54,55,56].

Isoform 1 dysfunction is associated with a number of metabolic diseases that result from the apparent deficiency of cortisone reductase. As a result of increased cortisol metabolism, the HPA axis is overstimulated, and its feedback inhibition is impaired, which in turn results in increased secretion of cortisol and adrenal androgens. The decreased ratio of the sum of cortisol metabolites to the sum of cortisone metabolites in the urine may manifest in women with hirsutism, acne, menstrual disorders, infertility and polycystic ovary syndrome (PCOS) [34,44,57,58,59,60]. 11β-HSD1 expression in adipose tissue cells promotes the development of abdominal obesity, which in turn is associated with serious consequences: type 2 diabetes, hypertension, dyslipidemia and other cardiovascular complications [34,44,57,58,59,61]. The impaired function of 11β-HSD1 in the central nervous system may affect the occurrence of degenerative diseases, including Alzheimer’s disease [44,58,59,60]. 11β-HSD1 may also be involved in the expression of inflammatory cytokines in keratinocytes and play an important role in exacerbation of atopic dermatitis (AD) by modulating the local availability of GCs [62,63].

The action of GCs is highly context-dependent and can vary widely during acute and chronic inflammation. 11β-HSD1 is induced early during the inflammatory response and shapes its subsequent trajectory. 11β-HSD1 deficiency/inhibition worsens acute inflammation, while 11β-HSD1 inhibition reduces inflammation in obesity or atherosclerosis. This is because physiological concentrations of GCs are not harmful and have anti-inflammatory effects; while chronic stress has an immunosuppressive effect, GCs inhibit Th1 functions and increase Th2, leading to a proinflammatory state [64]. Chronic inflammation in obesity is probably the cause of 11β-HSD1 overexpression, especially in metabolically active adipose tissue. 11β-HSD1 overexpression increases the level of cortisol, promoting growth of adipose tissue, and the excessive amount of adipose tissue increases the concentration of 11β-HSD1. In experimental studies, overexpression of 11β-HSD1 in visceral adipose tissue of transgenic mice promotes increased exacerbation of all of the disorders characterizing MetS [65], whereas mice deficient in 11β-HSD1 activity (knock out) were found to be resistant to stress and hyperglycemia caused by a fatty diet [66].

Compared to 11β-HSD1, 11β-HSD2 was not found in adipose tissue, and its activity is probably not related to obesity or MetS. 11β-HSD2 inactivates GCs, preventing activation of the mineralocorticoid receptor. The 11β-HSD2 mutation (reduced activity of 11β-HSD2) causes a rare syndrome of apparent mineralocorticoid excess (SAME) associated with arterial hypertension, hypokalemia, and fluid retention [60,67,68]. SAME is a genetic disease. 11β-HSD2 in the placenta is an important enzyme determining the proper development of the fetus; the disturbance of the activity of this enzyme correlates with low birth weight and a predisposition to the occurrence of hypertension, hypokalemic alkalosis and glucose intolerance [58,69,70]. Hypokalemia may cause arrhythmia, nephrogenic diabetes insipidus and rhabdomyolysis; together with hypertension, it may contribute to increased mortality. Hypokalemic patients excrete few tetrahydro-metabolites of cortisone (THE); the urinary free cortisol/cortisone ratio, which perhaps most accurately reflects renal 11β-HSD2 activity, is dramatically elevated [60,67]. AME (apparent mineralocorticoid excess) also plays a key role in the etiology of secondary arterial hypertension; it is assumed that AME may contribute to the development of approximately 30% of all cases. Hypertension, if untreated, results in damage to organs such as the kidney, cardiovascular system, retina and central nervous system [70]. Symptoms similar to AME, despite normal 11β-HSD2 activity (enzyme inactivation—substrate saturation of 11β-HSD2), can also be observed in the case of excessive cortisol production in Cushing’s syndrome or ectopic ACTH secretion [60,68,71].

The protective effect of 11β-HSD2 against the appearance of the features of MetS has been proven in experimental studies. Resistance to the development of diet-induced obesity, lower fat mass, lower food consumption, higher energy consumption, better glucose tolerance and insulin sensitivity were observed in transgenic mice having increased expression and activity of 11β-HSD2 in adipose tissue [72].

## 4. Disorders in the Function of 11β-HSD1

11β-HSD1 is highly expressed in the key metabolic organs: liver, adipose tissue, skeletal muscle, and the islets of Langerhans; therefore, disorders in the function of this 11β-HSD isoform are mainly connected with the occurrence of diseases and metabolic disorders. 11β-HSD2, in turn, has a role in blood pressure regulation. Its inhibition or genetic deficiency causes apparent mineralocorticoid excess and hypertension due to inappropriate glucocorticoid activation of renal MR [73]. In animal studies, e.g., in obese Zucker rats, 11β-HSD1 activity was decreased in liver but enhanced in adipose tissue [74]. However, the results of animal studies are not the same in all obesity models. What was described, among others, was a polygenic model of MetS, which demonstrated adipose tissue glucocorticoid deficiency but selective liver glucocorticoid amplification [75]. 

Studies concerning obesity in humans also confirm abnormalities in 11β-HSD1 regulation in adipose tissue [76]. This enzyme regenerates cortisol from cortisone; therefore, enhanced activity of this enzyme leads to the increase in cortisol concentration in local tissue. Active GCs bind with glucocorticoid receptor in glucocorticoid target tissues such as adipose tissue and liver [77]. In obese males, more rapid conversion of [(3)H]cortisone to [(3)H]cortisol in abdominal subcutaneous adipose tissue (SAT) was demonstrated [78]. Local increase in GC concentrations in tissues entails activity similar to Cushing’s syndrome, i.e., inducing hyperglycemia and insulin resistance, and consequently obesity exacerbation [79] despite the fact that the concentration of GCs in blood plasma does not change (which is characteristic for Cushing’s syndrome) [74,80]. Increase in 11β-HSD1 activity was demonstrated in SAT in obese persons [73,74,81,82], whereas in liver of obese persons, 11β-HSD1 activity decreases [74] or remains the same [83]. What was observed, among others, was that 11β-HSD1 activity in liver is the same in thin persons as in persons with excess weight/obesity without diabetes and in persons with excess weight/obesity with type 2 diabetes [83]. Decrease in activity of this enzyme in the liver of persons with visceral obesity may serve as a defense mechanism, preventing further body mass increase and glucose intolerance [53,84].

It has not been unequivocally explained, so far, whether in people with obesity, 11β-HSD1 activity increases also in visceral adipose tissue (VAT). Difficulties are probably a consequence of the fact that it is not easy to indicate activity of this enzyme locally, in real time. According to Alfonso et al. [85], the presence of intracellular hypercortisolism in VAT in human obesity is possible but unlikely. Tomlinson et al. [86], for instance, proved that expression of 11β-HSD1 in whole adipose tissue (SAT and VAT), adipocytes, or preadipocytes is not increased in human obesity. Neither were statistically significant correlations observed between 11β-HSD1 mRNA and body mass index. Alberti et al. [87], in turn, demonstrated higher expression of 11β-HSD1 in VAT than in SAT; however, VAT 11β-HSD1, in contrast with SAT, was not associated with metabolic disorders. Other authors have proved that enhanced regeneration of cortisol in SAT, and not in VAT, is connected with visceral obesity occurrence in females [88]. The authors demonstrated higher 11β-HSD1 activity in VAT than in SAT, but the 11β-HSD1 mRNA level in both tissues did not differ. They also observed a positive correlation between 11β-HSD1 mRNA level in SAT and VAT volume [88]. 

11β-HSD1 acts as an important regulator of inflammation [77]. Studies of recent years demonstrated that fructose consumption leads to metabolic changes, the effect of which, apart from adiposity and insulin resistance, is chronic low-grade inflammation [77,89]. Inflammation leads to the increase in 11β-HSD1 activity [77]. What influences 11β-HSD1 activity are proinflammatory mediators: tumor necrosis factor α (TNF-α) and interleukin-1 (IL-1) [90], which enhance the expression of this enzyme in human adipose tissue [91]. Enhanced 11β-HSD1 expression, in turn, stimulates the expression of TNF-α and interleukin-6 (IL-6), which proves that the increase in GC concentration aids inflammatory reaction [18]. 11β-HSD1 activity is also influenced by the level of H6PDH, which provides NADPH, indispensable for the course of reduction reaction catalyzed by 11β-HSD1 [92]. It was demonstrated that a diet rich in fructose enhances H6PDH expression [93]. Inflammation may be a key link between obesity and obesity-related disorders, such as hypertension, insulin resistance, dyslipidemia and diabetes [94]. Correlations were demonstrated between hepatic and VAT 11β-HSD1 expression with dyslipidemia and insulin resistance [95].

Together with body mass increase, the risk of type 2 diabetes occurrence increases [96]. Studies confirmed that in persons with type 2 diabetes, intracellular cortisol exposure is increased. It was demonstrated in these persons that both the urinary tetrahydrocortisol (THF) + allo-THF)/tetrahydrocortisone (THE) and cortisol/cortisone ratios were higher than in healthy persons. The ratio (THF + allo-THF)/THE indicated in urine is the measure of whole body 11β-HSD activity, whereas the cortisol to cortisone ratio reflects 11β-HSD2 activity [60,97]. A significant disorder of 11β-HSD activity was demonstrated in persons with diabetic kidney disease [97]. Shukla et al. [98] observed higher 11β-HSD1 activity in diabetic persons than in healthy controls. Studies also confirmed higher 11β-HSD1 activity in SAT in obese persons and in persons with type 2 diabetes than in healthy persons without diabetes [82]. Visceral obesity and type 2 diabetes are the components of MetS, which is the main factor for cardiovascular disease development [99,100,101,102,103,104]. It was proved both in animals and in humans that alterations in 11β-HSD1 activity in adipose tissue and liver are associated with MetS [105]. 

A cardiac visceral fat consists of the epicardial adipose tissue (EAT) located within easy reach of the coronary vessels and the mediastinal adipose tissue (MAT) located away from the arteries. In addition, the epicardial adipocytes, except their own intrinsic inflammatory activity, attract macrophages that impair the vascular functions by triggering inflammation and injuries [106]. Although the mRNA expression levels do not necessarily correlate with the levels of encoded protein and/or its enzymatic activity [107,108], Atalar et al. presented promising experimental results supporting the view that GCs acting on the MAT contributed locally to the development of coronary atherosclerosis, one of the causes of coronary artery disease (CAD) [109]. This study, with participation of 31 obese patients with CAD and 16 obese patients without CAD (controls), indicated that plasma cortisol level was significantly (*p* = 0.006) higher in the CAD group compared to controls. Intracellular analyses of MAT and EAT biopsies collected during coronary artery bypass grafting (obese CAD patients) have shown significantly (both *p* = 0.026) higher levels of stearidonic acid than that measured in control sample biopsies collected during heart valve surgery. Moreover, the mediastinal adipose tissue was characterized by the highest mRNA expression levels of 11β-HSD1 and GR, as well as almost 2-fold higher CD68 (macrophage marker) mRNA levels compared to EAT and SAT in obese CAD patients. MAT and SAT derived from obese CAD subjects have significantly (*p* < 0.05 and *p* < 0.001, respectively) increased expression levels of 11β-HSD1 mRNA vs. obese controls. Multiple linear regression analysis has demonstrated the association between stearidonic acid and mRNA expression levels of 11β-HSD1 and GR (R^2^ = 0.402). Patients suffering from CAD have revealed a positive moderate correlation (according to [110] between MAT expression levels of mRNA of 11β-HSD1 and GR (r = 0.529, *p* = 0.001) [109]). In another study, Atalar et al. demonstrated that the CAD group did not differ in terms of the plasma cortisol level, but MAT mRNA expression levels of 11β-HSD1, GR, and CD68 were significantly (*p* < 0.05) higher in sample biopsies derived from CAD patients (n = 37) than from non-CAD controls (n = 19). The authors showed a positive correlation of weight and 11β-HSD1 expression (r = 0.624, *p* = 0.014), as well as abdominal fat volume and GR expression (r = 0.437, *p* = 0.032) among CAD patients. Furthermore, a moderate correlation exists between CD68 and 11β-HSD1 mRNA expression levels (r = 0.410, *p* = 0.039), which was also confirmed by the protein expression levels determined immunohistochemically [111]. In turn, other data indicate that mRNA expression levels of adrenomedullin (a vasodilative peptide with a protective effect on the vasculature and the heart [112]) were significantly (*p* < 0.05) higher in extracts of epicardial adipose tissue obtained from CAD patients (n = 12) than in subjects without CAD (n = 10). Moreover, they were all positively correlated with 11β-HSD1 mRNA expression levels [107]. On the other hand, the results obtained by Al Bakir et al. were more ambiguous. They made an attempt to find a relationship between the whole blood 11β-HSD1 mRNA levels (measured by RT-qPCR method) and risk factors for development of cardiovascular diseases, such as alcohol, smoking habits, and duration of menopause [113] (a lack of protective effect of estrogen on the cardiovascular system [114]). The circulating 11β-HSD1 mRNA levels in smokers were 1.4-fold lower than in nonsmokers (*p* = 0.048), whereas higher alcohol consumption has been associated with increased systemic 11β-HSD1 mRNA levels. Moreover, the longer the duration of menopause, the lower the 11β-HSD1 mRNA levels (r = −0.422, *p* = 0.008) that were observed [113]. 

The analysis of tissue 11β-HSD1 mRNA levels, as well as peripheral concentrations of cortisol/cortisone and/or their tetrahydrometabolite (THM) ratios, do not directly reflect the actual activity of this enzyme. However, Baudrand et al. have found that patients with MetS showed normal levels of cortisol and cortisone in plasma/urine and significantly higher levels of urinary THMs (αTHF (*p* = 0.017), βTHF (*p* = 0.016), THE (*p* < 0.001), total THMs (*p* < 0.001)) than patients without MetS. In a logistic regression model, the levels of GCs metabolites were associated with dyslipidemia (OR 1.10, CI 95% 1.02–1.19), hyperglycemia (OR 1.09, CI 95% 1.01–1.18), and hypertension (OR 1.12, CI 95% 1.04–1.21) [115]. 

A comprehensive Mendelian randomization study assessed the role of cortisol-related pathways in CVD development [116]. Single nucleotide polymorphisms (SNPs) in human genes were analyzed in relation to the higher morning fasting plasma/serum cortisol. The following genome-wide association studies (GWAS) were considered: CORNET consortium (12,597 subjects), Shin et al. (7824 subjects), as well as Long et al. (2049 subjects). Other SNP data sets were also investigated regarding the occurrence of CAD, ischemic stroke, and type 2 diabetes mellitus (T2DM) derived from CARDIoGRAM-plusC4D 1000 Genomes-based GWAS (cases = 60,801; controls = 123,504), MEGASTROKE (cases = 40,585; controls = 406,111), and DIAMANTE (cases = 74,124; controls = 824,006). Genetic associations responsible for adiposity, higher blood pressure, and wrong glycemia and lipid profiles were also taken into account. The authors indicate that the role of the cortisol pathway in CVD needs to be better understood. Researchers also point to the relationship between stress and inflammatory factors in CVD. Nevertheless, the results described earlier show that this research area is interesting for further exploration and study.

## 5. Examples of Selective 11β-HSD1 Inhibitors as Potential Drugs Supporting the Treatment of Cardiovascular Diseases

Excess cortisol causes, among others, the appearance of symptoms characteristic of MetS, i.e., visceral obesity, insulin resistance, diabetes, dyslipidemia, hypertension and hyperuricemia [117]. MetS is a collection of abnormalities, of which insulin resistance, obesity, dyslipidemia, hyperglycemia, and hypertension are the major risk factors for type 2 diabetes and cardiovascular disease. [118]

It is well-known that intracellular cortisol concentrations are determined not only by plasma levels but also by the activity of 11β-HSD1, which catalyzes the conversion of inactive cortisone to active cortisol, especially in the liver and adipose tissue [119]. Inhibiting the activity of 11β-hydroxysteroid dehydrogenase type 1 reduces the level of cortisol, and thus, by reducing the mass of adipose tissue, insulin resistance and central obesity [120] or lowering the level of total cholesterol [121], helps to reduce the risk of cardiovascular diseases.

In recent years, significant activity in the academic and pharmaceutical community has led to the discovery of many new chemical compounds as specific inhibitors of 11β-HSD1. Selective inhibitors have significant potential as a pharmacological treatment for type 2 diabetes, obesity and cardiovascular disease [122,123].

Carbenoxolone is the known inhibitor of 11β-HSD1 (Figure 3). It is the hemisuccinate ester derivative of glycyrrhetinic acid, a natural product found in licorice root. Not only does carbenoxolone inhibit 11β-HSD1, but it is also—to a lesser degree, though—an inhibitor of 11β-hydroxysteroid dehydrogenase type 2 [124]. Inhibition of 11β-HSD2 may lead to hypertension by activation of the mineralocorticoid receptor in kidneys, as well as by decreased gene expression of endothelial nitric oxide synthase. This is particularly important to patients with type 2 diabetes and other metabolic syndrome elements [125]. In addition, the excess of mineralocorticosteroids and alleged hiperaldosteronism caused by taking carbenoxolone can cause peripheral edema, hypokalemia, and metabolic alkalosis. Non-selective activity of carbenoxolone affects its limited range of clinical applications, prompting a search for new compounds—selective inhibitors of 11β-HSD1.

**Figure 4:**
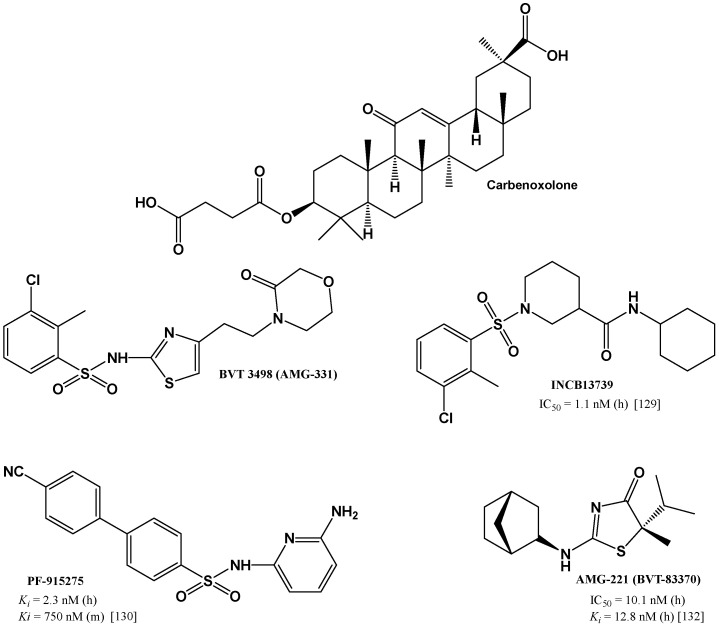
Carbenoxolone and selected selective 11β-HSD1 inhibitors used in clinical trials: BVT-3498 (AMG-331), INCB13739, PF-915275, AMG-221 (BVT-83370). Data refer to tests for human (h) or murine (m) 11β-HSD1 [129,130,132].

In the last two decades, many compounds have been synthesized and tested in the search for selective 11β-HSD1 inhibitors. Some of them have reached various phases of clinical trials (Figure 4). The first selective 11β-HSD1 inhibitor that was tested in clinical trials was BVT-3498 (AMG-331). The compound was in phase II clinical development and later terminated [126]. Unfortunately, the literature lacks information on the exact values that would allow the assessment of the inhibitory activity of this compound. The Biovitrum patent only provides the information that “BVT3498 has *K_i_* value for 11β-HSD1 in the nanomolar range” [127]. INCB13739 of Incyte Corporation was well tolerated in healthy volunteers and the patients with type 2 diabetes. After 12 weeks, 200 mg of INCB13739 resulted in significant reductions in A1C, fasting plasma glucose, and homeostasis model assessment-insulin resistance (HOMA-IR) compared with placebo. Total cholesterol, LDL cholesterol, and triglycerides were all significantly decreased in hyperlipidemic patients. Body weight decreased relative to placebo after INCB13739 therapy [128,129].

Phase I clinical trials were carried out, among others, for PF-915275 from Pfizer and AMG-221 (BVT-83370) from Biovitrum and Amgen [126,130,131,132]. 

Despite clinical trials, due to their discontinuance for unknown reasons in different phases, it has not been possible to introduce any of the tested compounds as a drug so far, hence the need for a further search for selective inhibitors. Further research on the synthesis and 11β-HSD1 inhibitory activity increases the chances of selecting new candidates for clinical trials.

**Figure 5:**
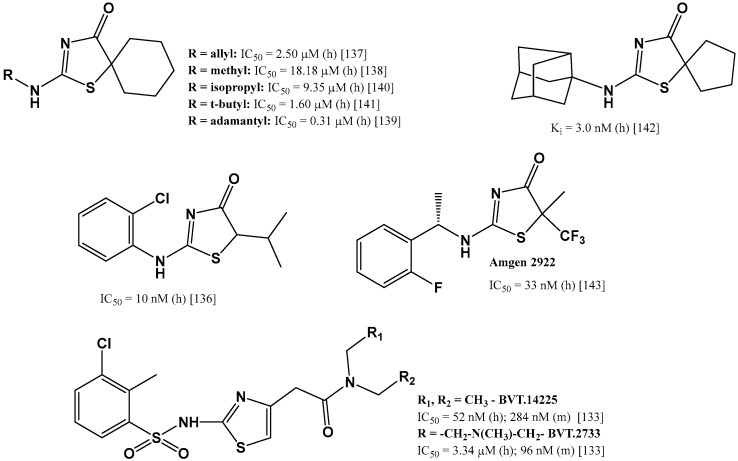
Selected selective 11β-HSD1 inhibitors with thiazole and thiazol-4-one moiety in in vitro studies. Data refer to tests for human (h) or murine (m) 11β-HSD1 [133,137,138,139,140,141,142,143].

**Figure 6:**
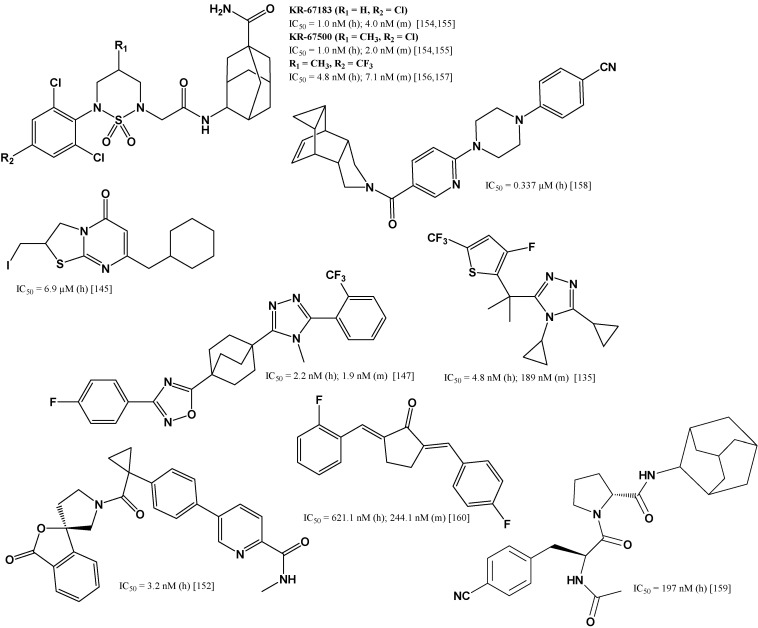
Selected selective 11β-HSD1 inhibitors with different heterocyclic moiety in in vitro studies. Data refer to tests for human (h) or murine (m) 11β-HSD1 [135,145,147,152,154,155,156,157,158,159,160].

The biological activity of a compound depends on its chemical structure, hence structural fragments characteristic for inhibitors that have reached the stage of clinical trials are observed in the new tested compounds. In vitro studies were carried out for various structures, of which the group of compounds containing a thiazole ring [117,133,134,135] or its partially hydrogenated form, thiazol-4-one [136,137,138,139,140,141,142,143], seems to be the most numerous and promising (Figure 5). Compounds containing fused systems with a thiazole ring [144,145] and other various heterocyclic systems with nitrogen atoms [146,147,148,149,150,151,152] were also analyzed as potential selective inhibitors of 11β-HSD1 (Figure 6).

Many compounds that inhibit the activity of 11β-HSD1 contain a sulfonamide moiety [122,144,153]. For example cyclic sulfonamides are highly selective inhibitors [154,155,156,157]. In many tested compounds with high activity and selectivity, the adamantyl substituent [139,146,149,150,151,153,154,156] and other polycyclic condensed systems [142,158,159] as well as halo-substituted phenyl rings [133,136,143,154,160] are constituents of the structure (Figure 6). 

## 6. Summary

In recent years, idiopathic obesity has reached the status of an epidemic in highly developed countries. It is often accompanied by disorders such as insulin resistance, hypertension, dyslipidemia and carbohydrate disturbances. They are components of metabolic syndrome (MetS). In turn, MetS is an important factor in the development of cardiovascular diseases, which are one of the main causes of death. Therefore, it seems appropriate to fully understand its relationship with cardiovascular diseases, which will contribute to the development of effective therapeutic treatments for this disease. In addition, it is believed that all changes that are included in MetS are caused by, among others, an increase in adipose tissue mass. Adipose tissue plays an important role in the endocrine system, being not only a source of hormones, but also a place of their metabolism, which applies mainly to steroids. Adipocytes also produce various enzymes that are involved in the synthesis of steroid hormones. Produced in large amounts by adipocytes, 11β-hydroxysteroid dehydrogenase (11β-HSD1), which is involved in the local production of cortisol, may also play a significant role in the development of MetS and its cardiovascular complications. The association of 11β-HSD1 with obesity, insulin resistance, type 2 diabetes, dyslipidemia, inflammation and arterial hypertension makes this enzyme an attractive object of research and a target in the pharmacotherapy of civilization diseases. The current reports on the beneficial effect of reducing the activity of 11β-HSD1 mean that attempts are being made to search for its inhibitors for therapeutic purposes. Research conducted over the last 20 years has shown that the most attractive compounds contain the thiazole ring and its hydrogenated form. The presence of appropriate substituents (including large hydrophobic cyclic systems) at this ring contributes to increasing the inhibitory activity and selectivity of the compounds in in vitro studies. This provides an opportunity to select representatives for clinical trials in the near future.

## Figures and Tables

**Table 1:** Direct comparison between the characteristics of 11β-HSD1 and 11β-HSD2 [44].

	11β-HSD1	11β-HSD2
Enzyme family	SDR superfamily	
Size gene	30 kb, 6 exons	6·2 kb, 5 exons
Size of the enzyme molecule	292 aa, 34 kDa	405 aa, 44 kDa
Enzyme kinetics	In vitro bidirectional Oxidation of cortisol to cortisone (11β-dehydrogenase)	Only dehydrogenase
	Reduction of cortisone to cortisol (11-oxoreductase) In vivo mainly reductase	
High affinity	Low for cortisol (Km-µM) High for cortisone (Km-nM)	High for cortisol (Km-nM)
Cofactor	NADP(H)	NAD
Tissue expression	Liver, brain, pancreas, adipose, lungs, gonads, bones	Kidney, colon, salivary glands, placenta
Function	Supplies cortisol to GR	Protects MR from cortisol

## Data Availability

Not applicable.
